# Discomforting surplus: gender, sexualization, and omissions in ethnographic fieldwork

**DOI:** 10.3389/fsoc.2023.1154435

**Published:** 2023-06-09

**Authors:** Phung N. Su, Phi Hong Su

**Affiliations:** ^1^Department of Sociology, University of California, San Diego, San Diego, CA, United States; ^2^Department of Anthropology and Sociology, Williams College, Williamstown, MA, United States

**Keywords:** gender, sexual harassment, embodiment, data surplus, positionality

## Abstract

As sisters and sociologists, we shared an unnerving experience of sexual harassment in one of our preliminary field sites. Our research pursuits split thereafter, with one of us leaning into questions of gender and sexuality and the other steering clear. Despite our diverging interests, we both encountered discomforting moments that raise questions about what data we render surplus in our analysis. In this article, we draw on ethnographic and interviewing data from our respective projects to conceptualize “discomforting surplus” as ethnographic data that we omit from our analyses. We offer two types of discomforting surpluses: those that reveal dissonance between our actions and self-conceptions, and those that seem not just uncomfortable, but inconsequential. We mine these discomforting surpluses, calling for introspection about our subject positions and the potential benefits of trying out analytical frames we have ignored. We conclude with practical suggestions for reflecting meaningfully on our relationships to the field and engaging in thought experiments that center discomforting surplus. These contradictions, omissions, and unnerving questions in ethnographic research are important to grapple with as we encounter a push for greater transparency and open science.

## 1. Introduction

Hoping to avoid the “ethnographic fixations” (Hanson and Richards, 2019) on solitude and danger, we traveled together to Berlin for the first time in July 2013. Phi was undertaking preliminary fieldwork for a dissertation in sociology, and Phung was about to begin her doctoral training in the same discipline a month later. We are sisters who grew up in the same household and studied in the same grade and, often, the same courses, until college. As siblings, we largely shared a subject position, a relational set of characteristics that included young (in our mid-20s at the time), Vietnamese American, bilingual (English and Vietnamese, with faltering German), heterosexual, and cis woman. We had also spent time in Germany in 2004 as part of a high school study abroad program. As teenagers, we experienced Germany as profoundly transformative, a respite from the intergenerational, intercultural tensions and unsettledness of our working-poor migrant household in a Los Angeles suburb. Nearly a decade later, we excitedly plotted our return to Germany.

We thwarted solitude on this much-anticipated trip, but danger found us. Phi was interested in relations between Vietnamese who had arrived in former East Germany as contract workers and those who had come to the former West as refugees. Because of our southern Vietnamese, forced migrant backgrounds, Phi anticipated having a trickier time gaining access to former contract workers, whom the media depicted as northern (Schubert, 2004). We therefore planted ourselves in the eastern part of the city near Dong Xuan Center, a bustling wholesale market where Vietnamese former contract workers and new arrivals congregated. “Bustling,” though, is in the eye of the beholder. Though Phi had read descriptions to that effect, the market and the surrounding district of Lichtenberg felt to us inert: Dong Xuan Center fell outside of the railway that circled Berlin's core, and the buildings in the east appeared to us a series of undifferentiated concrete slabs. In contrast to the throngs of tourists around Alexanderplatz, the area around Herzbergstraße appeared deserted as we later exited the gates of the large market complex. Against this gray backdrop, with few signs of life save for the occasional metal humming of the tram passing through, we were stalked in broad daylight for several blocks.

Though the exact details elude us now, our pursuers appeared to be a group of young men, some with shaved heads gesturing lewdly and catcalling us. Realizing they intended to approach us, we broke with our typical leisurely pace. Practically leaping across the empty street, we hoped our chasers would give up, but their echoing jeers trailed us down the block. As petite women (4′11” and 5′4”) in a city we could not fully navigate in the native language, we panicked. We ducked into a gas station but, lacking the vocabulary to explain our situation and with no excuse for loitering, we reluctantly headed back outside. There, the men had been waiting at a distance, and continued their chase. We sped up again and turned off Herzbergstraße, uncertain of what to do should they catch up with us. Hearing their voices booming around the corner we had just turned, Phi frantically grabbed a loose brick sitting atop a pile of rubble on the curb. Wielding it belligerently in her hand, Phi recalls shouting, “I'm gonna fucking bash their heads in!” With Phi's death grip on the brick, we rushed the remaining few hundred meters back to our hotel.

Recounting this confrontation proved difficult—and here's the rub—because Phi, intending to abandon her project after repeated instances of sexual harassment during this trip, disregarded the cardinal ethnographic task of recording notes. Instead, we offer this timeline of events from messages we shared in writing with others much later and from reconstructing our route from Dong Xuan Center to the gas station and our (no longer operational) hotel using Google Maps. Yet unrecorded does not mean forgotten.

Long after the adrenaline and fear dissipated, our discomfort remained. Discomfort at having felt weak because of our size. Discomfort at being unable to seek help because of our insufficient language skills. And, most frustrating of all, discomfort at being confronted with feelings of powerlessness in the face of sexual harassment. The trip would spark an ongoing conversation of our (mis)adventures as gendered, sexualized, and racialized bodies moving through space. However, these vexing experiences and our subsequent conversations about them seemed extraneous to the research because Phi was interested in ethnic politics and community formation, not in gender and sexuality.

Though Phi would eventually revive her research in Berlin, she left that initial visit wary of situations obviously imbued with sexualization; meanwhile, Phung leaned into questions of gender, sex, and mobility. Our shared encounter of sexual harassment in Phi's field site motivated Phung's exploration of sexuality in relation to reproduction and women's bodies. Significantly, we both selected field sites where we were not readily marked as racial others. Phi focused on cultural and religious organizations attended predominantly by Vietnamese migrants, and Phung would eventually immerse herself in the study of brides from rural Vietnam to Taiwan and South Korea, in the process also engaging with the Vietnamese men they left behind. To some degree, our site selections mitigated the racialized aspects of sexual harassment, though, as we discuss later, our national differences continued to mark us. Yet because of her topic of study, Phung frequently had to humor sexualizing comments and gazes in conversations with matchmakers, marriage brokers, and other male interlocutors. She rehearsed a gendered role rooted in non-threatening femininity and submission to gain access to potential gatekeepers. Doing so meant she felt viscerally the “costs of conducting the kind of ethnography that does not conform with feminist expectations” (Hoang, 2015: 192). But rather than treating this as a split of our true and false selves, we take this opportunity to explore how discomforting situations in the course of fieldwork reveal situational selves, including selves we may not recognize (Verdery, 2018).

In this article, we consider how uncomfortable ethnographic encounters—henceforth, discomforting surplus—can deepen our analyses. Our starting point is Joan Fujimura's awkward surplus, those “unanticipated research results that experimenters [in studies of sex genes] recognized as problematic or awkward and that they thus ignored in their final conclusions” (2006, p. 51). As a framing device, awkward surplus invites us to critically reexamine our claims. Hanson and Richards (2019) have usefully deployed awkward surplus to highlight sexual harassment during ethnographic fieldwork. Yet for two reasons that we elaborate below, we distinguish the data surplus of interest to us from what Fujimura considered awkward surplus. First, we offer discomforting surplus to better aid the analysis of ethnographic, rather than experimental, data. Second, we consider how the inclusion of such surpluses might well strengthen, rather than always undermine, our initial analyses.

With an eye toward the growing call for transparency and open science, we find that exercising reflexivity is crucial for advancing our understandings and practices of ethnographic fieldwork. In our case, we recognize that the projects we subsequently pursued and how we selected our field sites bear the imprint from this episode in Berlin, shaping the questions we asked and the social environments we could comfortably enter. Because we are not alone in pursuing research topics and navigating fieldwork with the remnants of personal experiences and encounters directing our analytical vision, we draw explicitly on prior contributions in the section that follows. In the conclusion, we reflect on how patterned omissions in ethnographic fieldwork pose challenges and possibilities for the push for open science.

## 2. Discomforting surplus

We are interested in what data ethnographers omit, as data surplus is built into our research methods. For example, social scientists drawing on large datasets do not make full use of every variable or potential correlation. To do so, particularly before establishing hypotheses, would constitute data mining. It is for this reason, among others, that researchers increasingly offer pre-analysis plans before collecting or beginning to analyze data. Yet cases that do not fit researchers' hypotheses do not disappear into the void; they continue to inform regression analyses. By contrast, ethnographers and interviewers not only share the data surpluses that quantitative researchers do, but the researcher who uses qualitative methods “also produces the data, such that the data collector is explicitly in the data themselves” (Small and Calarco, 2022, p. 12). Ethnographers and interviewers affect social interactions through our wording and mere presence. Because we are part of the data, they more obviously reflect our interests than, say, a battery of questions posed by the World Values Survey. Our concern here is therefore not *that* data surplus exists in qualitative research, but *how* such surplus might be patterned. Hence, we need to interrogate which data we discard or ignore if we are to take seriously the call for more transparency in research.

Here, we look to Fujimura's (2006) concept of awkward surplus, which she mobilizes to explain discrepancies in scientific experiments that sought to isolate sex genes in mice. Fujimura argues that scientists ignored data that challenged their initial assumptions or hypotheses, including instances in which the supposedly male-determining gene *Sry* actually resulted in more fertile female mice (2006 p. 51). The concept of awkward surplus offers three contributions:

“…first, to help us attend to unanticipated results that are recognized as problematic or awkward by experimenters and are thus ignored in their conclusions. Second, the concept provides an opportunity to reexamine unexpected experimental results either by using different frames or perspectives or by reexamining them in conjunction with data from other sources. Third, the examination of awkward surpluses provides a space where scientists and social scientists can work together in the production of new knowledge” (Fujimura, 2006, p. 71).

Sociologists Hanson and Richards (2019) have fruitfully applied the concept of awkward surplus in *Harassed: Gender, Bodies, and Ethnographic Research*. They observe that as “calls for reflexivity have become more common, they have paradoxically resulted in only superficial acknowledgment of the effects embodiment has on fieldwork” (2019, p. 154–5). The authors offer suggestions toward the valuable goal of reducing sexual violence in ethnographic fieldwork, starting with rejecting the “ethnographic fixations” on danger and intimacy, recognizing all research as embodied, and revamping ethnographic training to center that recognition.

We welcome Hanson and Richard's application of awkward surplus to ethnographic methods, but extend and refashion the concept as discomforting surplus for two key reasons.

First, discomforting surplus refers to the embodied experiences of inquiry in the social sciences, whereas awkward surplus refers specifically to experimental STEM contexts. We find that transporting the concept of awkward surplus as such to the social sciences sidesteps what Fujimura saw as a key contribution, which is to allow for collaborations across the natural and social sciences. For Fujimura, this contribution is crucial because debates on topics such as “the biology of sex is too important to leave to biologists alone because they usually are not trained to attend to and analyze how sociocultural frames influence their own experimental processes” (Fujimura, 2006, p. 74).

Rather than stretch the concept of awkward surplus and disregard its call for dialogue across the natural and social sciences, we draw on our experiences as sociologists to show how intradisciplinary brainstorming can similarly illuminate discomforting moments. Interdisciplinary collaborations within the social sciences may do so as well: one example comes from sociologist Ulrike Bialas and anthropologist Jagat Sohail addressing what they acknowledge to be an uncomfortable question: “If [migratory] flight is so traumatic, how can refugees fantasize about repeating it” (2023, p. 9)?

Second, discomforting surplus might well strengthen instead of contradict our initial analyses, in contrast to Fujimura's awkward surplus. One example comes from a discomforting moment that nearly became surplus. In *Divided by Borders: Mexican Migrants and Their Children*, Dreby (2010) recounts witnessing an interlocutor, Efrén, “take a swing at [his wife, Claudia] in front of [the guests]” (2010, p. 58). Efrén then followed Claudia into another room and repeated his transgression. After consoling the couple's oldest son, who had witnessed this violence, Dreby spoke at length with Efrén and “said that he should not worry and that [Dreby] would forget about the whole incident for [her] book” (2010, p. 58). In this instance, an ethnographic event can be discomforting if its disclosure harms interlocutors reputationally or emotionally. At Efrén's insistence, however, Dreby included the incident. Dreby mobilized this instance to illuminate gender roles and the family tensions exacerbated by restrictive border regimes. The discomfort that nearly became surplus ultimately deepened rather than countered Dreby's analysis. But what about when ethnographic events are rendered surplus because they pose discomfort or harm to the researcher(s)?

Building on Hanson and Richards, we take as our starting point our shared experience of sexual harassment. To borrow from Kathy Davis and Janice Irvine, “our own stories have shown us that silences, neglected feelings, and blind-spots can beset virtually all research areas” (2022, p. 6). The “stigma of *feeling* our research and feeling *bad* about our research” forms what Ghassan Moussawi refers to as “bad feelings” (2021, p. 78–9). Similar to “bad feelings,” our concept of discomforting surplus rejects the binary of “field/non-field… [a separation that] does violence to people's embodied and temporal experiences of research, and reinforces notions of disembodied, privileged researchers” (Moussawi, 2021, p. 80). As a case in point, our shared experience of sexual harassment in Berlin did not stay confined to the physical space of the city. Instead, Phung carried those embodied reminders to later field sites in South Korea, Singapore, and Vietnam. Phi also transported her heightened anxiety of public spaces back home to Los Angeles. There, she made a habit of carrying pepper spray, even in the neighborhood where she and Phung grew up.

Our discomforting feelings were thus not just internal, but social and relational, affecting how we experienced spaces and situations (citing Ahmed, 2004; Ngai, 2007; Moussawi, 2021). These experiences imprinted us in discomforting, rather than simply awkward, ways because they defied and redefined how we previously navigated public space and how we understood ourselves. And we rendered them surplus by omitting them from our subsequent analyses. What follows is our attempt to excavate these discomforting surpluses.

Discomforting surpluses may be patterned in a number of ways; here we offer just two: moments that highlight dissonance between our self-conceptions vs. actions, and moments that we assume are tangential to our key interests. The first maps onto discussions of ethnographers' subject position in the relational fields that constitute our research sites. The second offers a different type of thought experiment—what if I lean into this angle I had discarded, bringing this thread from background to foreground? What does that do to and for my analysis? We elaborate these by first revisiting Phung's performance of passive femininity to highlight discomforting surplus around subject position. We then explore Phi's avoidance of a gendered framing to demonstrate discomforting surplus we deem insignificant for our analyses. Both instances of discomforting surplus remind us of the importance of transparency in our analyses of our own subject positions and experiences in the field.

## 3. Methods

This article draws on field notes from our respective projects, each on Vietnamese migration but with different empirical questions, interlocutors, and sites. The ethnographic and interview data from Phung's work on gendered patterns of outmigration from rural Vietnam come primarily from her time in two countries, Singapore and Vietnam (the Mekong River Delta region and Ho Chi Minh City). Ethnographies and interviews were conducted between June 2014 to August 2014 and August 2017 to December 2018. In Singapore, Phung conducted ethnography and interviews at two bride market agencies. In Vietnam, she spent time at various locations, such as interlocutors' workplaces, homes, cafes and restaurants in the city, in addition to visiting interlocutors' family homes in the Mekong River Delta region. From there, she carried out semi-structured interviews with 62 interlocutors, 56 men and 6 women. Interviews covered various topics, such as the Vietnamese bride market, interlocutors' personal histories, views about Vietnamese women and gender norms, international marriages, work, and migration experiences. Phung conducted the interviews, which often lasted between 45 min to 2 h, in Vietnamese and English (in Singapore). With her interlocutors' permission, she recorded the interviews, which she transcribed and translated within 2 days from when they took place. Identifying information was edited out to protect her interlocutors' privacy. All names provided in the findings are pseudonyms.

Phi's discussion draws on data from ethnographic fieldwork in Berlin in Summer 2013, Summer 2014, and Fall 2015 to Summer 2016. She engaged in participant observation at two Vietnamese cultural organizations and three Buddhist pagodas. Additionally, she conducted 81 semi-structured in-depth interviews, largely in Vietnamese with some smatterings of English and German. She voice-recorded a majority of interviews and took notes by hand in instances in which interlocutors did not consent to being recorded. Further, Phi shadowed key interlocutors across various aspects of their social lives, sitting with them at their workplaces, translating for them at medical offices, and sharing conversation over home-cooked meals. She has anonymized the data that appears in this article to protect her interlocutors' confidentiality.

## 4. Results

### 4.1. Performing unassuming femininity: discomforting surplus that challenges self-conceptions

Among the things Phung left out in her dissertation are the doubts and tension about her own self-conception and how it is tied to the changing forms of power, privilege, and vulnerabilities individuals experience in fieldwork. As Victoria Reyes tells us, researchers carry an ethnographic toolkit, which “consists of researchers' social capital and backgrounds, among other characteristics, and shapes field access, field dynamics, and data analysis” (2020, p. 221). Ethnographers' visible and invisible toolkits can be marshaled to secure data, at times through solidarity and at other times with ambivalence. Shared experiences can facilitate access in ethnographic fieldwork and interviews, as demonstrated by Enriquez (2020) and López (2022), both spouses in mixed-status marriages. Life experiences and roles that we do not consider paramount to our research can also inform how we build trust. This is demonstrated in Nadia Y. Kim's rapport with her environmental justice co-organizers. It was not her organizing background, but her “willing[ness] to endanger [her] baby's health to partake in the movement” that seemingly earned Kim the organizers' trust (Kim, 2021, p. 169). In addition to gaining access, our subject positions also shape what we notice. For instance, Gowri Vijayakumar's new role as a mother during fieldwork animated her attention to mothering in her analysis of sex work in Bangalore (Vijayakumar, 2022).

The following encounter between Phung and the owner of a bride matchmaking company in Singapore illustrates how she leveraged her relational ethnographic tools to gain access, yet more pressing and perhaps unbeknownst to her is how Phung's analysis of the interplay between masculinity and femininity for her project was informed by the steps she took to access the field and the social scripts she rehearsed to stay in it. It illustrates Phung's ambivalence and doubt about the self-narrative she believed she embodied. As with other interactions throughout her field work that were discomforting and, as a consequence, eliminated from analysis, this example demands further contemplation about the situational selves that we assume in the field (Verdery, 2018).

I am dressed in a light pink chiffon dress and sit with my legs crossed at the ankles, a picture of demureness. My face is lightly powdered, not too much, but enough to hide any blemishes and project an image of flawlessness and paleness. Opposite me sits Travis, the owner of a matchmaking agency in Singapore. This is my second visit to his agency. Travis tells me about how Singaporean men who come to his agency want Vietnamese women who are skinny and big-chested, proclaiming, “I [am] like Starbucks, high and good quality of class. I don't want low quality.”

I press further to ask him what is considered “good” quality and Travis leans back in his chair, his hands cupping the back of his head as he smiles indulgently at me. His eyes wander up and down my body and settle on my chest for a slight second before settling on my face. He smirks and leans forward, resting his arms on the table to tell me. “Women who look like you. You understand? Men will pay a lot for you.” I laugh off his comment in an effort to maintain cordiality. Travis's grin widens as he looks back at me.

Travis approves. He orders his assistant to get some contracts from the drawer to show me how they conduct business. He hands me a copy of the contract with enthusiasm and tells me to keep it for my research. Now that he sees I would not repudiate him for the comments he made about me and Vietnamese women, Travis starts to tell me more about the operations of the bride market in Singapore.

Phung's field notes: Singapore, August 7, 2014

Although she felt inwardly repulsed and conflicted, Phung knew that her ability to secure a copy of the matchmaking contract relied on her performance as a gendered subject. She enacted a form of femininity that is rooted in the heteropatriarchal expectations of womanhood that Travis deemed appropriate: meek and wide-eyed. It was through this performance that Phung could learn about the workings of a bride market agency and the ideologies about gender, race, and nationhood that shape the preference for Vietnamese brides. In other similar moments, Phung often spoke submissively, always referring to herself by *em*, a pronoun that identifies a younger woman in the company of older people. The pronoun usage in Vietnamese communicates and demands adherence to hierarchies around age, gender, and social status. As such, casual exchanges between Phung and her interlocutors were imbued with hierarchy, as when a younger male interlocutor adopted the pronoun of older brothers (*anh*) as he urged her, “...you should come back and talk to me.”

Beyond interrogating the cultural orthodoxies that might limit our field interaction and inform our view of participants, bringing in discomforting surplus means purposefully integrating certain elements of our selves that come into conflict in the field. These “embodied costs” (Hoang, 2015) exemplify a dilemma between our participation in the field sites and the ways we behave within them that do not comport with our feminist sensibilities (Avishai et al., 2013; Hanson and Richards, 2019). This leads to questions such as: How do I reconcile my feminist politics with my performance of gender in the field? Am I who people think I am, even if they have misjudged me in some crucial way (Verdery, 2018)? Engaging, rather than shying away from reflecting on such questions, might have sharpened Phung's awareness of her positionality and made visible the unrecognized power and privilege, as tied to nationhood, that researchers have at their disposal. And doing so explicitly, in the body of her writing, would have allowed readers a fuller sense of the scene.

Had Phung included analysis of the toolkits she utilized in ethnographic field work, it would have clarified the assumptions Phung made about rapport as well as the limits of shared experiences. Initially, Phung believed that rapport would be automatic because of the ethnicity she shared with her interlocutors. But in fact, Phung's assumptions impeded her from recognizing Glenn's (1999) insight that we ourselves are carriers of unequal power as we tread through different cultural landscapes. That is, individuals can be part of the majority in one setting and members of the minority in another, and vice versa. This was made abundantly clear during Phung's first attempt to enter a place of business in Ho Chi Minh City that employed primarily men from the countryside. It is worth noting that, having accompanied Phi on her first entry into the field in Germany and experienced sexual harassment alongside Phi, Phung elected to bring a companion to her field sites. This came in the form of her then-unemployed and recent college-graduated cousin, who was born and raised in Vietnam and joined Phung especially when she spent time in male-dominated spaces.

“The men are in the back napping,” Tuan, the owner of VT Gas, tells me. I make my way into the kitchen with his wife and my cousin. Tuan's wife announced to the group of workers, about nine to ten men in their 20s and 30s who are dressed in navy jackets with the name of the company embroidered on their left chest pocket, that I want to interview them. Before she could finish her sentence, the men, who were lounging in the kitchen, started to push one another out of the way in a race to run as fast and as far as possible out the back door. The phrase, “They are scattering like flies” comes to mind as I stand there, mouth agape. They've made their way to the outside, and many are leaning against the wall, some pulling out cigarettes and lighting each other's buds. I asked why they did that and they yelled out different versions of “I'm shy/embarrassed,” “I don't know how to talk to women.” Tuan's wife explains that “Rural men without wives don't know how to talk to girls,” especially educated girls from abroad.

Phung's field notes: Ho Chi Minh City, November 28, 2017

Given that Phung's dissertation project centered on human mobility, weaving this discomforting surplus into the analysis would have revealed insights about unequal nationhood and who has the ability to participate in spatial mobility. Though this group of men was willing to talk to Phung's cousin, they grunted and shook their heads at Phung despite countless requests from all three women for them to speak with her. Reflecting on this moment now shows how individuals on the move, such as ethnographers, must confront the ways their experiences of minoritization in one setting might not carry into another. This is particularly pertinent to those whose positionality locates them at the intersection of multiple oppressions in the West. Illuminated in this encounter is the disparity that exists between Phung and her potential interlocutors, men with limited education from poor, rural origins who were employed as manual laborers. Her surprise at the men's behavior highlights her limited understanding of the boundaries of respectability that were made so sorely palpable during this scene. Her inability to conceal her gender, nationality, and education further underscores a moment of rupture between Phung's assumed familiarity with the men because of her ethnicity and the recognition that she was an outsider with a markedly different set of economic and cultural capital. The power differential between researcher and interlocutors is neatly captured in this image of the men standing on the outside of the house instead of resting in the kitchen during their break while Phung remained in the kitchen, a figure of disturbance and strangeness.

Leaning into this discomforting surplus and embedding it in analysis of nationhood would illuminate how power differentials between individuals take shape within shared territories and across different national boundaries. Phung's field notes detailed the countless times that her interlocutors brought up her identity as an American “overseas Vietnamese” (*Viet Kieu*), such as when one man claimed that he wanted a “*Viet Kieu* girl from the United States to come and marry [him]” or when Vietnamese women in South Korea and Taiwan remarked on how an American *Viet Kieu* inspires more awe from Vietnamese people than a Taiwanese or Korean *Viet Kieu*. However, these moments did not find their way into Phung's analysis. Similar to Phi, who focused on ethnic nationhood to the exclusion of gender and sexuality, Phung's analytical centering on gender and sexuality came at the expense of attention to the privileges that individuals embody as a result of unequal nation-state dynamics. Hence, this discomforting surplus illuminates how nationhood and citizenship shape perceptions of women's desirability.

Finally, we suggest that mining discomforting surpluses can alert us to how ethnographers measure privileges as well as possible dangers and vulnerabilities in field research. Here, we build on thoughtful reflection on the researcher in ethnographic fieldwork (Rios, 2011; Cobb and Hoang, 2015; Hoang, 2015; Small, 2015; Reyes, 2018; Hanson and Richards, 2019; Shulist and Mulla, 2022). For example, in 2014, Phung's initial research plan was to study the Vietnamese bride market phenomenon, which necessitated that she be in conversation with various actors such as brides, grooms, their families, government officials, and matchmakers. Commercial matchmaking is prohibited in Vietnam, and as such, Phung's access to the matchmakers was contingent on a government official, Binh, an important gatekeeper to access to the bride market in Vietnam, who was working outside the remit of the law. An encounter with Binh ultimately led Phung to shift her research focus.

Binh, a government official in Vietnam, promises to get me access to a house of girls (*nha nuoi gai*). He says that that is where there will be plenty of women and matchmakers to interview. I tell him that I will have my cousin come along with me but Binh shakes his head. He says it would be better if it were just the two of us, so that others would not get suspicious. He proceeds to tell me that he would introduce me as his younger sister or younger woman (*em*), that if I enter any space at his side, I would be able to speak to anyone I want. I insist that it would make me feel more comfortable to have my cousin who can balance my lack of knowledge about Vietnamese phrases and accents. Binh waves this off and tells me not to worry, he would be with me. During this conversation, Binh ignores my cousin, Hien, who has been by my side the whole time. He only speaks to me.

Phung's field notes: Ho Chi Minh City, August 26, 2014

Although we deploy discomforting surplus to clarify ethnographic analyses, the example with Binh reveals how analysis is necessarily intertwined with initial access and ongoing data collection. Because Binh was a well-connected and influential individual involved in the bride market in Vietnam, his promise to grant Phung behind-the-scenes access to the workings of matchmaking agencies was exciting. But more urgent was Phung's concern about safety if she were to be left alone with him. Phung ultimately declined Binh's offer. Her unwillingness to go along with Binh's suggestion and his refusal to have anyone else accompany them meant that she lost an important means of connection to a key population for her study of the bride market, sparking a series of adjustments to her research design. By ceasing communication with Binh, Phung forestalled potential discomforting surplus. At the same time, her maneuvers to prevent harassment meant that the specter of discomfort to come informed her access, data collection, and subsequent analysis. Through this example, we encourage a more deliberate consideration of our complex and contradictory subject positions, and how they shape questions of safety that guide the type of power dynamics we fix our analytical lens onto. We can further recognize how risk assessments are tied to access as well as the ways in which the glorification of danger ignores important intersections of vulnerabilities that shape individual experiences in ethnographic fieldwork.

In sum, when we omit encounters in the field that compromise our self-conceptions or gloss over the adjustments we make to our research plans, this can create a misleading portrait of a distant observer telling an “objective” story. By bringing discomforting surplus into our discussion and analysis, we make clear that ethnography does not occur in a controlled setting. Phung's modification to her initial plans demonstrates how experiences that derail research or drain us actually elucidate the very ways that risk assessment and vulnerabilities mediate social relations. Further, in the process of accessing a field site or disengaging from it, we must view with wary eyes the endless praise granted to the “cowboy ethnographer” in dangerous situations (Hoang, 2015, citing Contreras, 2012). By contrast, discomforting surplus locates ethnographers, as social actors, at the nexus of gender, class, race, nationality, and ability, which can inform their level of vulnerability, safety, and purview (Hanson and Richards, 2019). By mining this surplus, we are able to better recognize the type of knowledge that receives recognition and the kinds of “truths” that are subsequently produced. By centering discomforting surplus in our ethnographic analyses, we can better understand the situational selves—selves that are full of ambivalence and contradictions—that we embody and that exist as glimpses into how individual proximity to power and privilege changes in different national contexts.

### 4.2. Rejecting—and reevaluating—key narrative frames: discomforting surplus that seems inconsequential

Whereas, Phung rehearsed a mild-mannered femininity that contrasted with her self-understanding, Phi comfortably inhabited that role in the presence of her Vietnamese interlocutors, who were largely elderly and female. Like her sister, Phi grew up in a Vietnamese-speaking household and social environment that relied on kinship pronouns to refer to self and others, rather than less hierarchical addresses such as friend (*ban*). Most of her interlocutors also referred to her through such second-person addresses as niece, older sister, or younger sister. Only rarely did interlocutors refer to Phi as “friend” and to themselves as an unmarked “I” (*minh*), a practice that tries to avoid conveying hierarchy (Sidnell and Shohet, 2013). For Phi and the majority of her interlocutors, the default kinship figures of speech rendered natural the hierarchies of age, gender, and status that organize membership in the nation as imagined family (Seol and Skrentny, 2009). Phi did take note of, for example, how interlocutors instinctively ushered her into gender-segregated spaces. Yet she did not analyze this further.

In deciding to resume research in Berlin, Phi strategized ways to reduce the threat of sexual harassment; she succeeded so much so that she came to regard her earlier encounters as simply bad luck. Phi relied on her plain self-presentation, marital status, and research sites to spare her further harassment. In her everyday life, Phi dressed conservatively and rarely wore makeup. Although Phi did not wear a wedding band, her interlocutors knew she was married and occasionally interacted with her partner when he joined her for outings. Phi also never went out without her trusty pepper spray. Moreover, Phi spread her time across two cultural organizations and three Buddhist pagodas that were attended nearly exclusively by Vietnamese border crossers. She made these adjustments in part because the influx of forced migrants from Syria in 2015–16 meant that the umbrella migrant-serving organization she initially intended to shadow no longer had capacity to accommodate volunteers who did not have Arabic-language skills. As a consequence of changes in her field sites, Phi dramatically reduced the time she spent outside of predominantly Vietnamese social spaces. These precautions succeeded so that, in time, Phi no longer had a visceral reaction to returning to places such as Dong Xuan Center—albeit largely in the company of her White partner.

But as she felt confident that her body was not subjected to a sexualizing gaze, Phi also neglected the ways that other women's bodies were. One example comes from a young woman Phi called Kim, who had arrived in Berlin just a few months before she and Phi met. Kim looked forward to refining her language skills and pursuing undergraduate studies. Like several other young women Phi would come to know, Kim “knew” that she would ultimately have to birth a child on German soil or marry to stay in Germany. Kim recounted knowing this before she went abroad, but hoped that she might delay what she saw as an inevitability so that she could study for as long as possible.

Others in the field site similarly “knew” young Vietnamese women's reproductive fate. During a car ride with a group of aunts and uncles from a cultural organization she had been observing, Phi was uncomfortable hearing them discuss how Kim would lose her figure as soon as she became a mother. They gossiped to the effect of, ‘Her waist is so nice now, but it'll explode after she gives birth.' Phi was not surprised by the interlocutors' comments about weight and ideal Vietnamese beauty; indeed, the aunts would often lovingly chide Phi for putting on weight during fieldwork even though their cooking and insistence on her overeating contributed. Instead, what caught her off guard was that despite being married and older than Kim, Phi did not receive the same messaging about when and why she needed to have children. Less than a handful of interlocutors pressed about why she had no children, and most simply assumed she would one day. But the aunts and uncles impressed a sense of urgency to and about Kim.

The reproductive imaginaries to which young Vietnamese women were subjected often became realities. In late Summer 2016, as Phi prepared to depart from Berlin, she invited Kim and two other international students, Xuan and Yen, over for a home-cooked meal. Kim, Xuan, and Yen reflected on how much they had changed socially and politically since leaving Vietnam a year ago. They daydreamed about what transformations and possibilities the following years might bring. By the next year, Kim and Yen, by then just barely entering their 20s, would discontinue their studies and bear children. Xuan would marry, with Phi and her partner serving as witnesses to the union. Shortly thereafter, Xuan became a mother. To be clear, we do not mean to imply that motherhood is something to be lamented. Instead, what is crucial is that these women expressed wanting something different for themselves, but understood reproduction as the only way to secure long-term residency.

These descriptive, narrative threads of gendered paths toward staying in Germany feature in Phi's field notes, but what would it mean to take seriously such discomforting surplus in the analysis? In the book that resulted from her dissertation, Phi focused on nationhood after border crossings. She argued that Vietnamese in Germany still identify as one ethnic (if unequal) nation, but have rejected the nationalistic principle that their shared nationhood requires a shared state to represent them. But if Phi had foregrounded Kim's, Xuan's, and Yen's gendered migration pathways in her previous analysis, she would have more convincingly revealed the ways that nationhood is stratified.

Specifically, Phi might have better recognized how such national stratification is read and expressed differently through the bodies of women. Here, Phi's ability to triangulate and compare with other women in the field offers valuable clues. It was not just education that mattered for how others interpreted women's reproductive obligations or lack thereof. Another woman, a White German PhD student, likewise built relationships with Phi's refugee interlocutors. Yet, like Phi, she did not recall pervasive comments about whether and when she should proceed to the next stage of a heteronormative reproductive life course.

It was also not just citizenship that mattered—or, at least, not just Phi's U.S. citizenship. A prime example comes from another interlocutor, Ina, who was a few years older than Phi and a German citizen daughter of Vietnamese refugees. During Phi's field work, Ina was expecting her first child. Though Ina's parents were excited, neither they nor she suggested that her first pregnancy was occurring unreasonably late in life. What mattered for Ina as well as for Phi was where they ranked in the ethnic nation. Ina's German upbringing seemingly gave her a pass in a context where the average age of first-time motherhood was over 30 years (Janjevic, 2022).

By virtue of being tethered to Western citizenships, Ina and Phi could exercise more autonomy in their reproductive choices without ubiquitous pressure. Because of her Vietnamese American background, Phi was assumed to embody the best of what the nation could offer: educational and economic successes, frankness and sincerity in her relationships (Su, 2022, Chapter 4). By contrast, women who were recent arrivals to Germany or who grew up in northern Vietnam were positioned lower in the ethnic nation. This meant that they were seen as mere vessels for achieving socioeconomic mobility through staying in Germany.

Though unintended, Phi's insistence on streamlined storytelling came at the cost of fuller transparency and reflection about how the construct of the nation is inherently gendered. In her book, Phi focused on how nationhood served as a central organizing principle in the everyday lives of Vietnamese border crossers in Berlin. Yet she saw this framing as demanding primacy over, rather than complementing, an analysis of gender. As a result, she missed the ways that a gender and sexuality lens could have productively amplified her argument by revealing how understandings of nationhood are mobilized through and enacted on women's bodies. Indeed, striking discrepancies emerge in Phi's field notes, as when an organization she called Refugees for Germany insisted that for the Lunar New Year celebration, women must don traditional silk dresses, whereas men were free to sport Western suits.

Even as she concluded her project, Phi recognized that her omission of an analysis of gender was all the more conspicuous because “state power, citizenship, nationalism, militarism, revolution, political violence, dictatorship, and democracy—are all best understood as masculinity projects, involving masculine institutions, masculine processes and masculine activities” (Nagel, 1998, p. 243). Yet Phi insisted that while she observed the actions of cis men and cis women, she did not study gender and its constitutive relationship with border crossings. But as this thought exercise suggests, the leap from description to analysis need not have demanded an entirely different narrative arc. Instead, an analysis of gender and sexuality beckoned from within the framework Phi offered, and had more to contribute to the analysis than its treatment as irrelevant, discomforting surplus would allow.

## 5. Discussion

We have demonstrated how data surplus can manifest through moments that illuminate tensions between our self-conceptions and actions as well as events or threads we assume to be inconsequential for our analyses. The first discomforting surplus highlights ethnographers' performances of gender, anxieties about vulnerabilities, and unrecognized privileges and power. Such instances remind us of the sociological insight that different situations produce versions of the self, including selves we might not readily recognize. The second discomforting surplus offers an opportunity to reexamine themes we might otherwise dismiss. It provides a generative platform in which we take seriously the oft-posed question, “What about X,” but offer a modification that invites further analysis: “How might the inclusion of X further my analysis of Y?” We consider these discomforting surpluses, augmenting Fujimura's concept of awkward surplus and building on the work that Hanson and Richards began to mine the insights of this concept for a social scientific and ethnographic audience.

Contradictions, omissions, and unnerving questions in ethnographic research are important to grapple with, particularly as we encounter a push for greater transparency and open science. Yet, as Small and Calarco (2022) contend, calls for open science and reproducibility ask us to judge qualitative methods by the standards of quantitative or experimental ones. Moreover, calls for transparency as an unqualified good ignore the vulnerabilities of the populations with whom many of us work (Bloemraad and Menjívar, 2022). Rather than full transparency, then, we encourage exercises to deepen reflexivity, particularly about data we might be inclined to silence. We arrive at this through demonstrating how deeper reflexivity regarding what we systematically omit can enrich our analyses.

We conclude with two practical suggestions, the first of which concerns when and how we reflect on our relationships to the field and its impact on our claims. Often, we see key, enlightening discussions of subject position exiled to the ends of monographs in a methodological appendix. We offer one example from a celebrated ethnography. Not until the very end of *Evicted: Poverty and Profit in the American City* does Matthew Desmond disclose that he is the “friend” who lent an interlocutor the money she desperately needed. He cites being motivated by the fact that “there is a bigger game afoot,” such that his interests lie “in a different, more urgent conversation” about housing policies and the persistence of inequality (Desmond, 2016, p. 335). We can appreciate the delicate dance between making transparent our impact on the site vs. making ourselves the story's center. Yet by removing ourselves from the storytelling completely, we obscure how our subject positions shape our data.

Our point is as much about *whether* as about *how* and *where* we reflect on our subject positions in relation to our analyses. One instructive example comes from Moussawi's fieldwork on LGBTQ formations in Beirut in the context of local, regional, and global politics. He reflects on initially trying to separate the affective from the analytical by keeping separate field notes (2021, p. 81–2) or confining the discussion of “bad feelings” to methodological appendices. But collapsing this distinction between his feelings of unsafety as a queer person in Beirut during episodes of publicized violence allowed him to center “‘the situation' not simply as a descriptor but as a theoretical intervention” (2021, p. 90). We therefore find that acknowledgment of our subject positions—indeed, of how all knowledge is situated (Haraway, 1988)—strengthens rather than detracts from our contributions.

Our second suggestion is to deliberately engage in conversation and thought experiments that center discomforting surplus. Dialogue can be intradisciplinary, as between us as two sister sociologists. It can also be interdisciplinary, as between Bialas and Sohai (2023) about their shared focus on forced migration in Berlin. Conversations do not need to be exclusive to academic circles, either. Those of us doing community-engaged research already do share our questions, thoughts, and writing with relevant audiences, and invite their contributions, hopes, desires, and points of contention. Talk of feelings matters as a first step to counteract how “rarely… feelings of shock, irritation, fear, boredom or, for that matter, amusement, excitement and delight find their way into the analysis itself” (Davis and Irvine, 2022, p. 1).

Our fateful brushes in Berlin in 2013 stayed with us long afterwards, intellectually and behaviorally. As Phung began her doctoral studies after that trip, she nursed an interest in the ways that women's bodies are commodified toward social mobility for individuals, families, and the nation. Phi avoided such a study, and only recently stopped clenching pepper spray in public. Though our reactions diverged, the discomforting surplus of our shared experience of sexual harassment nevertheless informed our interests and expressed disinterests. Calling attention to two ways these surpluses can be patterned, we invite others to reexamine the experiences that, though we render them invisible, still inform our interpretations. This reflexivity nods to calls for transparency while still recognizing the need to protect the confidentiality of our often-vulnerable interlocutors.

## Data availability statement

The original contributions presented in the study are included in the article/supplementary material, further inquiries can be directed to the corresponding author.

## Ethics statement

The studies involving human participants were reviewed and approved by University of California Berkeley University of California Los Angeles. Written informed consent for participation was not required for this study in accordance with the national legislation and the institutional requirements.

## Author contributions

The authors confirm responsibility for the research design and data collection, to the analysis of the results and writing of the manuscript. All authors contributed to the article and approved the submitted version.
